# What distinguishes positive deviance (PD) health professionals from their peers and what impact does a PD intervention have on behaviour change: a cross-sectional study of infection control and prevention in three Israeli hospitals

**DOI:** 10.1017/S0950268820002484

**Published:** 2020-10-14

**Authors:** R. Cohen, A. Gesser-Edelsburg, A. Singhal, S. Benenson, A.E. Moses

**Affiliations:** 1 School of Public Health, University of Haifa, Haifa, Israel; 2 The Health and Risk Communication Research Center, University of Haifa, Haifa, Israel; 3 Department of Communication, The University of Texas at El Paso, El Paso, Texas, USA; 4 Inland University of Applied Sciences, Hamar, Norway; 5 Department of Clinical Microbiology and Infectious Diseases, Hadassah University Medical Center, Ein Kerem, Jerusalem, Israel

**Keywords:** Characteristics, infection prevention and control, positive deviance approach, socio-cognitive, social network

## Abstract

Past studies using the positive deviance (PD) approach in the field of infection prevention and control (IPC) have primarily focused on impacts on healthcare-associated infection rates. This research aimed to determine if health professionals who exhibit PD behaviours have distinctive socio-cognitive profiles compared to non-PD professionals, and to examine the impact of a PD intervention on healthcare professionals’ (HPs) behavioural changes in maintaining IPC guidelines. In a cross-sectional study among 135 HPs, respondents first filled out a socio-cognitive characteristics questionnaire, and after 5 months were requested to complete a self-reported behavioural change questionnaire. The main findings indicate that socio-cognitive variables such as external locus of control, perceived threat and social learning were significant predictors of a person exhibiting PD behaviours. Almost 70% of HPs reported behavioural change and creating social networks as a result of the PD intervention in maintaining IPC guidelines, 16.9% of them are a ‘PD boosters’ (a new group of HPs who have adopted the positive practices of PDs that were originally identified, and also added additional practices of their own). Social networks can contribute to internalizing and raising personal accountability even among non-PD professionals, by creating a mind map that makes each person believe they are an important node in the network, regardless of their status and role. Health intervention programmes should purposely make visible and prominent social network connections in the hospital system.

## Introduction

Rising healthcare-associated infections (HAI), resulting in high morbidity and mortality, represent a critical issue of investigation in global public health [1]. Despite widespread interventions and efforts, no institution or country claims to have solved this problem [2]. Hand hygiene compliance is reported to be suboptimal, HAI infection rates are high and often rising, and there is extensive resistance to change [3, 4].

Over the past three decades, while multiple intervention programmes have focused on increasing knowledge and awareness about reducing HAIs, the more innovative and effective ones have focused on behaviour models [5–7]. However, studies examining the effectiveness of behavioural interventions remain uncertain about the relative effectiveness of specific strategies, and it is unclear which of the combinations deliver the most effect [1, 4, 6, 8, 9]. In addition, Srigley *et al*. [6] in their review emphasised that behavioural models are more effective in raising compliance with infection prevention and control (IPC) guidelines, compared to interventions which only addressed knowledge and awareness. Therefore, there is a great need to integrate behavioural theory-based interventions with existing programmes to identify factors that motivate health care workers to adhere to IPC guidelines.

One of the socio-behavioural approaches that is gaining prominence in the field of IPC, especially in the past two decades, is the positive deviance (PD) approach. The PD approach is based on the premise that in every community there are individuals or groups whose uncommon behaviours and strategies enable them to find better solutions to problems than their peers, while facing worse challenges and having access to the same resources.

The PD approach is a method of grounded social inquiry along two dimensions. The first term, ‘positive’, refers to an action that optimises and improves a given situation, leading to a better solution for the same problem. The second term, ‘deviance’, refers to individuals who are exceptional on account of their uncommon (outlier) behaviour, which means that they differ from the majority and their normative actions [10]. PD differs from common approaches to problem-solving, as it seeks to identify and streamline existing resources derived from the staff within a setting, rather than import external ‘best practices’ (bottom-up) [11].

To date, studies of the PD approach in the field of infection control have focused on the effectiveness of the approach on HAI and hand hygiene compliance rates [12–17], and reducing gaps between guidelines and implementation [18, 19]. A before-after PD intervention study that was implemented in ICUs and non-ICUs across Veterans Affairs (VA) hospitals in the USA indicated a reduction in HAI of 62% (*P* < 0.001) in the rate of methicillin-resistant *Staphylococcus aureus* (MRSA) in ICUs and a reduction of 45% (*P* < 0.001) in non-ICUs [12, 13]. Similarly, a before-after PD intervention study in Billings Clinic in Montana, a healthcare organisation of repute in the USA patterned after the Mayo and Cleveland Clinic, reduced HAI (MRSA) infections by 84% between 2006 and 2009. The VA hospitals as well as Billings Clinic received high acclaim from CDC analysts for their highly significant statistical declines in their HAI rates on account of the PD intervention [20].

**Fig. 1.**
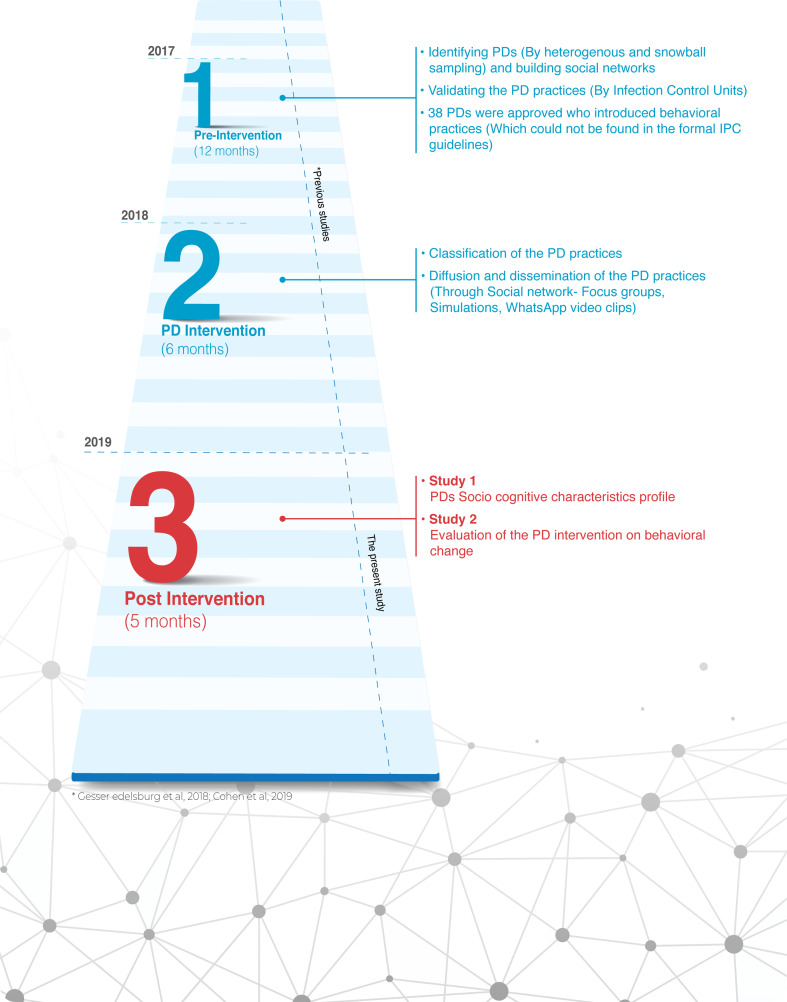
Flow chart of the quasi-experimental study.

Although it is known that the human factor in maintaining hygiene is the most influential in decreasing infection rates, so far, the behavioural component has not been examined in studies dealing with PD [1, 6, 7]. The present study is part of a larger quasi-experimental programme that was carried out over 2 years between November 2017 and December 2019 (Fig. 1). The overall aim was to implement the PD approach to identify HPs who exhibited unusual positive practices on the care continuum, to assess the effectiveness of those practices in reducing HAI rates and to disseminate those practices (as explained below).

The study has two specific objectives: first, to determine if professionals who exhibit PD behaviours have a distinctive socio-cognitive profile compared to other healthcare professionals (HPs) (study 1); second, to examine the impact of a PD intervention on behavioural change among these individuals in maintaining IPC guidelines (study 2), to allow a better understanding of the socio-cognitive capabilities of health professionals and the impact of PD on the behavioural outcomes of their peers. To the best of our knowledge, this is the first study identifying unique differences between PDs and their non-PD peer group of health professionals and the impact on behavioural change regarding IPC guidelines.

## Methods

### Framework

The quasi-experimental study included three phases: pre-intervention (12 months), PD intervention (6 months) and post-intervention (5 months). Our previous publications [18, 19] focused on the first two phases: The pre-intervention phase included the identification of PDs – that is, health professionals who practised non-normative (positively deviant) practices that delivered better outcomes in maintaining hygiene and (1) were not found on the formal IPC guidelines and (2) were scientifically validated by the infection control units – and mapping of their social networks [20]. The PD intervention phase comprised classification of the PD practices, followed by diffusion and dissemination of the observations.

The current study focusses on phase three, the post-intervention phase, divided into two sub-studies according to the objectives presented above. *Study 1 – probability of being a PD* (the cognitive profile of socio-PDs), conducted during July 2019. *Study 2 – impact of the PD intervention on HPs behavioural change*, conducted during December 2019.

In study 2, we focused on the average rate of the implementation of all PD practices that were identified and disseminated through the pre-intervention and after PD intervention phases; these were given the same weight as it is well known that infection prevention interventions call for a variety of guidelines and actions to be applied, and therefore variables can neither be isolated nor a determination made as to which practices are most effective. Moreover, this is considered a pragmatic study, thus one cannot evaluate randomised interventions due to ethical or logistic concerns [21, 22]. An additional aim was to examine whether the PD approach positively affects behavioural change in maintaining IPC guidelines, rather than comparing the efficacy of different practices.

### Research population

The research population included 135 HPs from five wards (two Internal Medicine and two Orthopaedics Departments, and one Medical Intensive Care Unit (MICU)), from three hospitals in Israel. The decision regarding the selection of the departments was made consultatively by the hospital management, the Infection Control Units, and the present researchers. The rationale was to select departments with a high incidence of HAIs and an accompanying commitment on part of the health professionals to collaborate on this study.

### Data collection

*Study 1* is a cross-sectional study that took place at the beginning of phase 3 (post-intervention), after a 12-month PD intervention period. A total of 135 HPs responded to a socio-cognitive characteristics profile questionnaire (Supplementary Material) via an online survey, using the Qualtrics XM platform, which was distributed via a WhatsApp group.

*Study 2* took place 5 months from the beginning of the post-intervention phase; 122 HPs responded to the self-reported behavioural change questionnaire, following the PD intervention. The decrease in the number of participants between the first and the second study was due to the dropout of HPs over time (e.g. moving to other wards, illness and maternity leave).

### Research tools

Study 1 – probability of being a PD (the cognitive profile of socio-PDs): The socio-cognitive characteristics profile questionnaire was first piloted and validated in a survey with 30 HPs [23–27] with adjustments (content validation) made to fit the research topic (infection control). We decided to focus on items that assess behavioural patterns and are found in the literature to be significant for decision-making processes and behavioural change. We did not include questions related to personality characteristics. Respondents were asked to rank their responses using a seven-point Likert scale, ranging from 1 (completely disagree) to 7 (completely agree), on statements under five components: locus of control, risk behaviour, fatalism, thinking style and social learning.

In the first part of the questionnaire, respondents were asked to provide their demographic and professional details, including their names, so we could compare PDs with those not identified as PDs. The second part included the following indexes:

1. Locus of control (LOC) includes six statements based on The Multidimensional Health Locus of Control Scale (MHLOC). The Internal LOC index was the average of three items, *α* = 0.55. For example, ‘The small actions I perform during my work, such as hand hygiene, have implications for the patient's life’. Likewise, the External LOC index was the average of three items, *α* = 0.66. For example, ‘Responsibility for the issue of infection control lies largely under the control of the health system’.
2. Risk behaviour diagnosis includes 12 statements based on the Extended Parallel Process Model (EPPM). Perceived Efficacy index was the average of six items, *α* = 0.80. For example, ‘If I maintain hygiene, the chance that my patient will get infected is small’. Perceived Threat index was the average of six items, *α* = 0.78. For example, ‘I believe acquired infections are a serious threat to patients’ life’.
3. Thinking style includes 10 statements based on Cognitive-Experiential Self-Theory (CEST). The Analytic Thinking index was the average of five items, *α* = 0.64. For example, ‘I prefer to do something that challenges my thinking abilities rather than something that requires little thought,’ and the Intuitive Thinking index was the average of five items, *α*  = 0.83. For example, ‘When it comes to medical care, I usually rely on my “gut feelings”’.
4. Fatalism includes eight statements about their fatalistic beliefs. A Fatalism index was the average of five items, *α* = 0.86. For example, ‘If patient acquired infectious disease, that is the way they were meant to die’.
5. Social learning includes two items based on social learning theory. The first demonstrates positive social learning, e.g. ‘When I do hygiene-related actions, I feel that the staff around me does the same’. The second demonstrates negative social learning, e.g. ‘In cases where I'm not strict about hygiene, I feel that people around me are also not strict’.

Study 2 – impact of the PD intervention on HPs behavioural change: In this evaluation study, the self-reported behavioural change questionnaire presented the list of the PD practices identified in their hospital ward, and then respondents were asked to rate (for each practice) the extent to which it changed their behaviour and motivated them to implement the practice in accordance with the following ratings: 1 – I did not implement, 2 – I implemented partially, 3 – I have fully implemented, 4 – I have fully implemented and added my own practices.

### Reliability and validity

Before the questionnaire was distributed, as noted previously, a content validation process was undertaken by a pilot study of 30 participants (10 participants from each hospital). The respondents were asked to provide feedback on the wording of the questionnaire, the length of the questionnaire and changes were made accordingly.

### Data analysis

Study 1: Probability of being a PD was tested using multiple logistic regressions, where PD was the dependent variable, and the indexes above were defined as independent variables along with three demographic parameters (gender, age and ethnicity). The *χ*^2^ tests were used to evaluate odds ratios. An *α* level of 0.05 determined statistical significance. All data were analysed using SAS 9.4 software (Cary, NC, USA).

Study 2: The impact of the PD intervention on HPs behavioural change was analysed using descriptive statistics. All practices identified at the three hospitals were pooled and the proportion of participants who rated each of the implementation levels was assessed.

### Ethics considerations and consent to participate

The study was approved by the ethics committee of the Faculty of Social Welfare and Health Sciences, University of Haifa, confirmation number 392/17. All the study participants gave their consent to participate in the research and to publish its findings. The research does not provide any medical or personal information by which each participant can be identified, thus anonymity is ensured.

## Results

### Sample description

A total of 135 HPs responded to the questionnaire (Table 1), 38 (28%) of the respondents were identified as PD. Most of the respondents were nurses 115 (85%), 12 (9%) were nurse assistants and 8 (6%) physicians.

**Table 1.** Respondents demographic characteristics (*n* = 135)

Characteristics	Category	*n* (%)
Gender	Male	39 (29%)
	Female	96 (71%)
Age (years)	Mean (range)	38 (22–65)
Sector	Nurse	115 (85%)
	Nurse assistant	12 (9%)
	Physicians	8 (6%)
Ethnicity	Jewish	82 (61%)
	Arab	53 (39%)
Seniority (years)	Mean (range)	11 (0.5–38)
Hospital 1	MICU	37 (27%)
Hospital 2	Internal Medicine Department	26 (19%)
	Orthopaedics	20 (15%)
Hospital 3	Internal Medicine	31 (23%)
	Orthopaedics	21 (15%)

### Study 1: probability of being a PD

Most independent variables showed no significant association with being a PD. Therefore, a reduced model (Tables 2 and 3) was used in a backward elimination process containing the following factors:

1. Gender (male): OR 2.97 (95% CI 1.20–7.36), i.e. odds of a man being a PD is approximately three times the odds of being a woman.
2. Perceived Threat: PDs perceived the issue of acquired infections as a real threat to patients’ life, with OR 1.83 (95% CI 1.05–3.18). Each unit increase in the perceived threat index increased the odds of being a PD by 83%.
3. LOC: PDs did not attribute the acquired infections to external causes that depend on the system (e.g. deficiency of standards, load), with OR 0.65 (95% CI 0.47–0.90). Each unit increase in the external locus of control index reduced the odds of being a PD by 35%.
4. Social learning: PDs perceived that if they did not take actions to maintain hygiene, this will have a negative impact on HPs who work alongside them who will also not take hygiene actions, with OR 1.36 (95% CI 1.09–1.70). Each unit increase in negative social learning increased the odds of being a PD by 36%.

**Table 2.** Predicted probabilities of being a PD: analysis of maximum likelihood estimates^a^

Parameter	d.f.	Estimate	Standard error	Wald *χ*^2^	Pr > *χ*^2^
Intercept	1	−4.5640	1.8509	6.0806	0.0137
Male	1	1.0882	0.4631	5.5213	0.0188
Perceived threat	1	0.6055	0.2819	4.6125	0.0317
External locus of control	1	−0.4361	0.1666	6.8550	0.0088
Negative social learning	1	0.3066	0.1148	7.1352	0.0076

a Logistic regression, reduced model.

**Table 3.** Four significant factors: odds ratio estimates of being a PD

Effect	Point estimate	95% Wald confidence limits	
Male	2.969	1.198	7.358
Perceived threat	1.832	1.054	3.184
External locus of control	0.647	0.466	0.896
Negative social learning	1.359	1.085	1.701

### Study 2: impact of the PD intervention on HPs behavioural change

During the PD intervention, behavioural practices were identified by 38 PD HPs, which could not be found in the formal IPC guidelines. The practices were on diverse topics on the patient's care continuum as follows: (1) removal and replacement of a dressing on a surgical cut; (2) removal of protective clothing when leaving an isolation room and performing hand hygiene; (3) procedure of taking a blood sample; (4) procedure of sending blood samples to the laboratory; (5) procedure of central line insertion; (6) washing a patient in bed; (7) sterilizing a stethoscope; (8) procedure of cleaning the patient's unit and surroundings; (9) taking a patient's urine sample with a urine catheter and sending it to the laboratory; (10) cleaning the nursing station; (11) mixing IV meds and carrying them to the patient; (12) replenishing disposable equipment in a patient room; and (13) instructing patients and families on maintaining hygiene in the hospital.

All practices were common to all units participating in the research except for the procedure of central line insertion that was specific to ICU.

The overall practice implementation ranked by all study respondents was 13.4% for not implemented, 17.2% partially implemented, whereas 69.4% rated the practices as fully implemented (52.5%) and implemented + additional practices (16.9%). In fact, the latest unexpected finding (implemented + additional practices) demonstrates that a new group has been formed, since they are not the original PDs identified at the beginning of the study. This new group is HPs who have adopted the positive practices of PDs that were originally identified, and also added additional practices of their own. Therefore, we coined the name and called them ‘PD boosters’.

## Discussion

This study was divided into two sub-studies. Study 1 sought to examine the probabilities of being a PD (the cognitive profile of socio-PDs), that is, what variables made PDs different from their peers? The findings indicated that there were no differences in thinking style and fatalistic beliefs among those identified as PD compared to other HPs.

The first difference that emerges is their perceived threat from acquired infections. PDs acted in line with their perception that acquired infections are a real threat to patients’ health, and probably understood that a hospitalised patient is exposed and vulnerable to acquired infections. The second difference is in external LOC, which means that PDs do not attribute the acquired infections to external causes that depend on the system (e.g. deficiency of standards, load). PDs profoundly understood that acquired infections are under their control and responsibility and therefore they take actions. The third difference is negative social learning impact. PDs perceived themselves as ‘influencers’ or ‘opinion leaders’ within their social network and realised that should they cease to act in accordance with the guidelines, this will have a negative impact on other HPs. These three key findings point to PDs displaying an enormous sense of accountability and responsibility to prevent infections.

According to Dohmann [28], accountability arises out of one's free choice and strong personal commitment to ensuring that a result is achieved. Additionally, he claims that a lack of accountability could result in poor practice. In contrast, it is anticipated that nurses who espouse professional accountability engage in life-long learning to maintain and enhance competence, promote quality patient care and uphold professional standards [29]. A systematic review of intervention programmes by Srigley *et al*. [6] concluded that interventions based on behavioural models were more successful in raising HH compliance than interventions that only addressed knowledge and awareness. The findings of the present study suggest another component that must be considered is developing and strengthening the sense of accountability in the field of IPC. We claim that the sense of accountability is the key element that distinguishes those who exhibit PD behaviours from those who do not. This claim underpins the deep understanding that everyone plays a role in preventing the transmission of infections.

Study 2 sought to examine the impact of PD intervention on HPs’ behavioural changes. The findings indicated that almost 70% of HPs reported full implementation of the PD, while almost 17% of them reported that they also added their own practices. Thus, they changed their behaviour following the PD intervention, and were encouraged to add positive practices to existing procedures on the care continuum. These findings are further strengthened when it comes to practices not found in the official IPC guidelines. An example of this was published by Cohen *et al*. [18], describing the process by which physicians from ICUs demonstrated PD practices while sequencing operations for central line insertion, which completed the missing parts of the official guidelines. These missing parts, called grey areas, encompass the variety of situations on the care continuum that are not addressed in accepted guidelines, and are where staff members are unsure of how to proceed; they interpreted or understood the guidelines differently [19].

The diffusion and social learning process was investigated by the construction of a social network map in the pre-intervention phase of the project, when staff members were asked to name other staff they believed to be PD, i.e. persons who demonstrated positive deviant behaviours to maintain HH or who raised ideas for such practices. Indeed, looking deeper into the structure of the social network [19], one can see that arrows indicating the internal connections within the network are mostly directed to the PDs. This observation corresponds with the literature in the field indicating that social learning from colleagues has a stronger impact, which means that people have a greater tendency to learn and adopt behaviours learned from their peers when guidance does not come from outside experts or above [30]. This reinforces the value of ‘social proof’ – the notion that if someone like me can do it, I can too. Furthermore, we observed there was a positive trend in behavioural change reported across all practices, therefore it can be concluded that the approach affected all levels and was not associated with a specific practice.

## Recommendation

When analysing the findings of the unique characteristics of the PDs alongside the success of the PD approach in driving behavioural change, every individual within the social network had an impact on the entire network. Especially in infection prevention programmes, we know that any action on the care continuum is essential in the prevention of infections, and therefore there is great significance for the engagement and accountability of all HPs through which the spread of acquired infections can be eradicated.

Considering the findings, our recommendations for future intervention programmes are:

1. Visibility and accountability through building social networks
  Building intervention programmes based on social network maps provides visibility to staff and may encourage their sense of accountability [1, 17, 31]. Moreover, as we observed that following the PD intervention, unexpectedly, some participants became a new group (‘The PD boosters’), as they not only fully implemented the practices, but also added their own riffs and tips. Therefore, increased their engagement and accountability and encouraged them to be more creative and contribute to the collective effort. Another interesting issue concerning the impact of the social network on the professional image of HPs was seen in the ongoing COVID-19 crisis. An increased media discourse on the spread of the virus and the importance of following infection prevention guidelines increased the professional role and value of the HPs in combating the virus [32–34].
  The World Health Organization (WHO) declared that ‘Health care-associated infections only usually receive public attention when there are epidemics. Although often hidden from public attention, the very real endemic, ongoing problem is one that no institution or country can claim to have solved, despite many efforts’ [2].
  The COVID-19 crisis ‘put on the map’ the HPs who soon became ‘heroes’ and ‘warriors’ in the eyes of all, and in return the staff demonstrated accountability and enlistment, without being asked by the system to do so. Therefore, focusing on intervention programmes that make visible the underlying social network of HPs is more likely to help the staff build and strengthen their sense of personal and collective accountability for eliminating healthcare-acquired infections. This reinforces the understanding that infections are the ‘problem of us all’ and not of the ‘system’ only.
2. HPs leaders for long-run social networks
  Health systems must act to identify, adopt and nurture their human capital by paying attention to building stronger personal and social networks within health systems, and identifying the most prominent people (leaders) through their multitude of connections on the social map to accelerate behaviour change and improve organisational performance over the long run [31]. The literature indicates that IPC interventions usually hold short term but fail over the long term [1, 35]. The social network manages to highlight people who may not have a prominent role in the institution's hierarchy, but their multiplicity of relationships with other people in the network is indicative of their strengths. Social network maps can show that an ordinary nurse may be an essential asset and resource through which health systems can assimilate, disseminate and maintain different practices over time [20, 36]. Therefore, the role of purposely and strategically mapping social networks within health systems must be further investigated.
3. Encouraging women's creativity within the social network
  Another finding of this study was that men were more likely to engage in PD behaviours, a function, perhaps, of gender socialisation that men enjoy a social legitimacy to take on proactive roles compared to women [37]. This finding is likely reinforced and explained by the fact that the health system management of the three hospitals under study was composed mostly of physicians and senior management who are male. Since 67% of participants who rated level 4 (fully implementation and additional practices) were women, it can be concluded that the implementation of the PD approach has stimulated creativity among these women. Therefore, intervention programmes also have a significant role in the gender aspect by encouraging women's creativity and giving them a more prominent role in the social network.

The study had some limitations, namely the sample size, as only five hospital wards in three hospitals with a total of 135 HPs were examined. Our results indicate that the research should be expanded to other hospital wards to examine additional components beyond those selected here. Second, in the socio-cognitive questionnaire, we asked respondents to give their names, so that we could make the comparison between those identified as PD and those who were not. Therefore, there is a concern that responses may have reflected social desirability. Data analysis showed that in some variables there was no variance between the two groups, so we assume that the respondents answered authentically.

In conclusion, HPs are clearly aware of the issue of infection prevention and take it seriously. The findings indicate that a visible rendering of social networks among HPs can contribute to increasing the sense of accountability even among non-PDs. Social network maps can create and generate a social and mind map that makes each person an important node in the network regardless of their status and role. Thus, if a person does not prevent the transmission of infections by their actions, the collective social and professional system is disbanded. HPs are empowered when the social network map is prominent and visible in the hospital system, so that each staff member perceives their contribution is recognised and appreciated by their colleagues and management. Moreover, according to the research findings, the social network will encourage people to be creative, because anyone can be a PD, anyone can contribute a tip or action that make a big difference in infection prevention.

## Data Availability

The data that support the findings of this study are available from Ricky Cohen, ricky.cohen83@gmail.com.

## Supplementary material

For supplementary material accompanying this paper visit http://dx.doi.org/10.1017/S0950268820002484. click here to view supplementary material
