# Supplementary material for: What distinguishes positive deviance (PD) health professionals from their peers and what impact does a PD intervention have on behaviour change: a cross-sectional study of infection control and prevention in three Israeli hospitals
Source: Epidemiol Infect. 2020 Oct 14;148:e259. doi: 10.1017/S0950268820002484 (PMC7689599; doi:10.1017/S0950268820002484)
Supplement: Supplementary file 1 [file S0950268820002484sup001.docx]

**Supplementary Material: Socio cognitive profile questionnaire**

The questioner will be a closed questioner, please mark the most appropriate answer. (There is no right or wrong answer).

In the next section there is a list of descriptive sentences that characterize different people, and they can be relevant to you or not relevant.

Check next to each description your level of agreement with the statement.

**Personal details and Professional status:**

Name

Age: (Years)

Gender: Male, Female

Ethnicity: Jewish, Arab

Sector: Nurse, Physicians, Nurse Assistant

Tenure: (Years)

**Risk behavior diagnosis scale (RBD)**

|  |  | Completely  Disagree- 1 | 2 | 3 | Neutral-4 | 5 | 6 | Completely  Agree- 7 |
| --- | --- | --- | --- | --- | --- | --- | --- | --- |
| 1) | Maintaining hygiene is effective in preventing acquired infections |  |  |  |  |  |  |  |
| 2) | Hygiene is working in preventing acquired infections |  |  |  |  |  |  |  |
| 3) | If I maintain hygiene, the chance that my patient will get infected is small |  |  |  |  |  |  |  |
| 4) | Maintaining hygiene during my work in the hospital is a convenient action |  |  |  |  |  |  |  |
| 5) | Maintaining hygiene during my work in the hospital is an easy action |  |  |  |  |  |  |  |
| 6) | I am able to maintain hygiene to prevent acquired infections |  |  |  |  |  |  |  |
| 7) | I believe acquired infections are a serious threat to patients' lives |  |  |  |  |  |  |  |
| 8) | I believe acquired infections cause significant illnesses |  |  |  |  |  |  |  |
| 9) | I believe acquired infections are a severe threat to patients' lives |  |  |  |  |  |  |  |
| 10) | Patients admitted to hospital are at risk of acquiring infection |  |  |  |  |  |  |  |
| 11) | It is likely that a patient admitted to hospital will acquire infection |  |  |  |  |  |  |  |
| 12) | Patient admitted to hospital are more susceptible to acquired infection |  |  |  |  |  |  |  |

**Rational-Experiential Inventory Scales**

|  |  | Completely  Disagree- 1 | 2 | 3 | Neutral-4 | 5 | 6 | Completely  Agree- 7 |
| --- | --- | --- | --- | --- | --- | --- | --- | --- |
| 13) | I don't like to think much about hygiene during my work |  |  |  |  |  |  |  |
| 14) | I avoid situations where I need to think deeply about how to maintain hygiene in complex situations |  |  |  |  |  |  |  |
| 15) | I prefer to do something that challenges my thinking abilities rather than something that requires little thought |  |  |  |  |  |  |  |
| 16) | I prefer complex to simple problems |  |  |  |  |  |  |  |
| 17) | Deep thinking about maintaining hygiene over time does not give me satisfaction |  |  |  |  |  |  |  |
| 18) | I trust my initial feelings about maintaining hygiene actions |  |  |  |  |  |  |  |
| 19) | I believe in the actions I take during patient care |  |  |  |  |  |  |  |
| 20) | My intuition about maintaining hygiene is usually correct |  |  |  |  |  |  |  |
| 21) | When it comes to medical care, I usually rely on my "gut feelings" |  |  |  |  |  |  |  |
| 22) | I can usually feel when the medical care I gave is right or wrong even if I cannot explain how I know |  |  |  |  |  |  |  |

**Locus of control**

|  |  | Completely  Disagree- 1 | 2 | 3 | Neutral-4 | 5 | 6 | Completely  Agree- 7 |
| --- | --- | --- | --- | --- | --- | --- | --- | --- |
| 23) | As a professional worker I am solely responsible for the patients' health |  |  |  |  |  |  |  |
| 24) | The small steps I perform throughout my workday, like hand-hygiene, have implications on the patients' health outcomes |  |  |  |  |  |  |  |
| 25) | The main thing that affects patient health depend on what I do |  |  |  |  |  |  |  |
| 26) | Responsibility for the issue of infections is largely controlled by the entire health system |  |  |  |  |  |  |  |
| 27) | No matter what I do, the conditions of the place will determine the health status of the patient |  |  |  |  |  |  |  |
| 28) | No matter what I do, God/destiny will determine the health status of the patient |  |  |  |  |  |  |  |

**Fatalism Scale**

|  |  | Completely  Disagree- 1 | 2 | 3 | Neutral- 4 | 5 | 6 | Completely  Agree- 7 |
| --- | --- | --- | --- | --- | --- | --- | --- | --- |
| 29) | If a patient is supposed to acquire infection disease, it does not matter if I maintain hygiene, they will be ill in any situation |  |  |  |  |  |  |  |
| 30) | If patient acquired infection disease, that is the way they were meant to die |  |  |  |  |  |  |  |
| 31) | No matter what medical staff do, if the patient was supposed to acquire infection, it will happen anyway |  |  |  |  |  |  |  |
| 32) | The patient will get diseases if they are unlucky |  |  |  |  |  |  |  |
| 33) | The patient's health is a matter of luck |  |  |  |  |  |  |  |
| 34) | I often feel helpless in dealing with problems of life |  |  |  |  |  |  |  |
| 35) | Anything related to acquired infections will not end well |  |  |  |  |  |  |  |
| 36) | There is really no way I can solve acquired infections |  |  |  |  |  |  |  |

**Social learning**

|  |  | Completely  Disagree- 1 | 2 | 3 | Neutral- 4 | 5 | 6 | Completely  Agree- 7 |
| --- | --- | --- | --- | --- | --- | --- | --- | --- |
| 37) | When I do hygiene-related actions, I feel that the staff around me will do the same |  |  |  |  |  |  |  |
| 38) | In cases where I am not strict about hygiene, I feel that people around me also are not strict |  |  |  |  |  |  |  |
